# Investigation of Anti-*Toxocara* and Anti-*Toxoplasma* Antibodies in Patients with Schizophrenia Disorder

**DOI:** 10.1155/2014/230349

**Published:** 2014-04-16

**Authors:** Shahram Khademvatan, Niloufar Khajeddin, Sakineh Izadi, Elham Yousefi

**Affiliations:** ^1^Health Research Institute, Infectious and Tropical Diseases Research Center, Department of Medical Parasitology, Ahvaz Jundishapur University of Medical Sciences, P.O. Box 613715794, Ahvaz, Iran; ^2^Department of Psychiatry, Ahvaz Jundishapur University of Medical Sciences, Ahvaz, Iran; ^3^Department of Psychology, Shahid Chamran University, Ahvaz, Iran; ^4^Research Institute for Infectious Disease of Digestive System, Department of Medical Parasitology, Ahvaz Jundishapur University of Medical Sciences, Ahvaz, Iran

## Abstract

*Objective*. The aim of the present study was to examine the relationship between *Toxoplasma gondii* and *Toxocara* spp. infections in patients with schizophrenia disorder. *Method*. A total of 100 patients with schizophrenia disorder and 95 healthy individuals participated in the study. Participants were tested for the presence of anti-*T. gondii* and anti-*Toxocara* spp. antibodies by ELISA and Western blotting. Data were analyzed using Chi-square test and Fisher**9**s exact test. *Results*. There were no differences in *T. gondii* IgG seroprevalence between patients with schizophrenia and healthy individuals (*P* = 0.1), but there were differences in seroprevalence between males and females with schizophrenia (*P* = 0.009). In contrast, *Toxocara* spp. IgG seroprevalence was greater in patients with schizophrenia disorder than in healthy individuals (*P* = 0.02), but there were no differences in seroprevalence between men and women with schizophrenia (*P* = 0.5). Finally, there were no differences in seroprevalence of *T. gondii* or *Toxocara* spp. IgG among different subtypes of schizophrenia, various age groups, residential area, or clinical course of treatment (*P* > 0.05). *Conclusion*. The present study suggests that patients with schizophrenia disorder are at elevated risk of *Toxocara* spp. infection. Moreover, contamination with *T. gondii* is a risk factor for schizophrenia in women.

## 1. Introduction

Schizophrenia is a severe, disabling mental disorder with a devastating impact on patients, their family, and society [1]. Schizophrenia is a heterogeneous disorder characterized by a range of clinical features such as positive and negative symptoms [2], including a reduction in patients' health-related quality of life [3]. The prevalence of schizophrenia has been reported to be 1% of the adult population [4], developing in late adolescence or early adulthood, and most patients suffer from the disease throughout their lifetime [1].

Toxocariasis is a helminthozoonosis, caused by the Ascaridida nematodes,* Toxocara canis* and* Toxocara cati* [5]. Dogs and cats are the definitive hosts of* T. canis* and* T. cati*, respectively, although other mammals, such as humans and rodents, can be infected [6]. Humans are infected by ingestion of infectious eggs [6], often as a result of direct contact with pets or consumption of contaminated vegetables or undercooked meat [7].* Toxocara* infection is also often transmitted by contact with the soil. Young children, people who live in rural areas, and people with soil-related occupation are at increased risk for toxocariasis [8–10]. The presence of* Toxocara *spp. larvae in the central nervous system may cause some neurological and psychiatric disorders including schizophrenia. Kaplan et al. [11] found more seroprevalence of* Toxocara* spp. infection in patients with schizophrenia than in healthy individuals (45.9% versus 2%). Further, in the study conducted by El-Sayed and Ismail [12], toxocariasis was detected in 23.3% of patients with schizophrenia disorder compared with that in 2.2% of healthy controls. In another study by Alvarado-Esquivel [13], 4.7% of psychiatric inpatients were positive for anti-*Toxocara* IgG antibodies compared with 1.1% of healthy controls. The difference was statistically significant.

Another parasite that is thought to be associated with schizophrenia is* Toxoplasma gondii*. Toxoplasmosis is one of the most common parasitic infections in human and other warm-blooded vertebrates including birds, livestock, and marine mammals [14, 15]. Humans usually become infected by consumption of undercooked meat, unwashed or poorly washed vegetables, or contaminated drinking water [16]. After a short phase of acute toxoplasmosis, the infection becomes latent and becomes encysted in the central nervous system and in muscle tissues, potentially for the life of the infected host [17, 18]. The parasite has the ability to alter the behavior of the host to increase transmission [19]. Some studies have found changes in infected individuals' personality characteristics [20–24].

There is much evidence to suggest that schizophrenia disorder is associated with toxoplasmosis [25–32]. Daryani et al. [33] and Hamidinejat et al. [34] from Iran also observed that higher titers of* T. gondii* IgG antibody was positively correlated with schizophrenia [33–35]. Some results however challenge the plausibility of this association [36, 37]. Because of these controversial findings and little knowledge about the prevalence of* Toxocara* spp. infection in patients with schizophrenia, the present study aimed to determine the seroprevalence of* Toxocara* spp. and* Toxoplasma* infections in patients with schizophrenia disorder and to compare it with healthy controls in Ahvaz, Southwest Iran.

## 2. Methods

Research was conducted over a period of 12 months from 2012 to 2013. A total of 100 patients diagnosed as having schizophrenia disorder and 95 matched healthy controls participated in the study. The patients had been admitted into Golestan hospital affiliated to Jundishapur University of Medical Sciences in Ahvaz, Iran. The diagnosis of schizophrenia disorder was performed by two psychiatrists following the DSM-IV-TR criteria. The control group consisted of blood donors who were tested at the laboratory in Jundishapur University of Medical Sciences. The controls were assessed by both a clinical psychologist and a psychiatrist and had no history of schizophrenia disorder. Neither of the 2 groups were immunodeficient nor were any other major psychiatric disorders or neurological diseases presentable.

To participate, subjects had to agree to comply with the requirements of study, and after describing the procedures and purposes of the study, written informed consents were obtained. The hospital's institutional review board approved all procedures.

A 5 mL blood sample was taken from each subject for serological analysis. Each subject was also asked to complete a questionnaire to obtain demographic data about ethnicity, gender, age, level of education obtained, marital status, and employment.

### 2.1. Serological Tests

Sera were separated by sample centrifugation at 3000 rpm for 5 min and then kept at −20°C until analysis. Anti-*Toxoplasma* antibodies (IgG and IgM) concentrations were measured by enzyme-linked immunosorbent assay (ELISA) (IgG and IgM: Trinity Biotech Captia, USA). In addition, all sera were examined by* Toxocara* IgG-ELISA test (IBL, International Gmbh, Hamburg, Germany) and* Toxocara* Western blot test (LDBIO Diagnostics, Lyon, France) for confirmation.

### 2.2. Statistical Analysis

Data were analyzed using Chi-square test, Fisher's exact test, and Student's *t*-test. Odds ratios (OR) with 95% confidence intervals (CI) were also determined. *P* < 0.05 was considered significant. Statistical analyses were carried out using SPSS v16 software.

## 3. Results

Demographic characteristics (sex, residence, marital status, level of education obtained, ethnicity, and age) of both patients and healthy controls are detailed in Table 1. Patient and control group seroprevalence for both* T. gondii* and* Toxocara* was evaluated. Anti-*Toxplasma* antibodies were detected in 34% of patients with schizophrenia disorder and 47.39% of healthy individuals (*P* > 0.05) (Table 2). Anti-*Toxoplasma* IgM antibodies were positive in 4 patients and 2 healthy individuals (*P* > 0.05).

Seroprevalence of anti-*Toxocara* IgG were higher in schizophrenia patients (14%) than in healthy controls (4.3%) (*P* = 0.02; Table 2). Western blot analysis confirmed* Toxocara* spp. infection, with low molecular weight bands (24–35 kDa) that are specific for* Toxocara* IgG present from all positive sera.

With respect to schizophrenia subtypes, anti-*T. gondii* antibodies were present in 35.82% (24 of 67) of patients with paranoid type of schizophrenia disorder, 42.85% (3 of 7) of patients with catatonic type, 33.3% (6 of 18) of patients with undifferentiated type, and 33.3% (1 of 3) of patients with residual type. None of the patients with disorganized type were seropositive. The difference in infection rate among the subtypes was not statistically significant (*P* = 0.9, Table 3). Anti-*Toxocara* antibodies were detected in 1.56% (10 of 64) of patients with paranoid type of schizophrenia disorder, 14.28% (1 of 7) of patients with catatonic type, 11.11% (2 of 18) of patients with undifferentiated type, and 20% (1 of 5) patients with disorganized type. None of the patients with residual type were seropositive. The difference in infection rate among the subtypes was not statistically significant (*P* = 0.5, Table 3). Serological analyses also confirmed that there were no significant increases in anti-*Toxocara* and anti-*Toxocara* antibody levels in patients with first-episode Schizophrenia disorder compared with those in patients with recurrent episodes (*P* > 0.05, Table 4).

The seroprevalence of anti-*T. gondii* IgG antibodies in male and female patients with schizophrenia disorder was 24.61% and 51.42%, respectively, (*P* = 0.009, Table 5). Anti-*Toxocara* antibodies were detected in 51.42% of females and 17.14% of males with schizophrenia disorder (nonsignificant). The seroprevalence of* T. gondii* infection in patients living in urban and rural areas was 21.05% and 16.66%, respectively, (nonsignificant, *P* = 0.7). Seroprevalence of* Toxocara* also was not different between patients living in urban and rural areas (Table 6).

The participants were divided into 5 groups based on their age (<20, 20–29, 30–39, 40–49, and >50 years). The seroprevalence of* T. gondii* infection in the five age groups were 0%, 32.25%, 40%, 26.08%, and 36.36%, respectively, in patients; in healthy individuals the seroprevalence was 0%, 44.57%, 71.42%, 0%, and 100%, respectively, (nonsignificant between or within age subgroups, *P* > 0.05, Table 7). For* Toxocara* seroprevalence, there were no differences between or within age subgroups (*P* > 0.05, Table 8). Anti-*Toxocara* antibodies were significantly more prevalent in men with schizophrenia disorder (12.30%) than in healthy men (1.92%) (*P* = 0.03), but there was no difference in women (*P* = 0.1).

## 4. Discussion

The present study was conducted to investigate associations between schizophrenia disorder and parasitic infections, toxocariasis and toxoplasmosis. Because of poor hygiene, the prevalence of* Toxocara* spp. is higher in most localities of Iran than any elsewhere in the world. Sharif et al. in northern Iran, Arbabi and Hooshyar in central Iran, Sadjjadi et al. in southern Iran, and Khademvatan et al. in Southwest Iran reported 44%, 13.3%, 52.8%, and 45%, respectively, [6, 38–40]. With prevalence of approximately 50% in Iran, toxoplasmosis continues to be a public health problem [41]. The present study is the first to report toxocariasis in patients with schizophrenia in the Iranian population. The association between* Toxoplasma gondii* and schizophrenia also has received little attention in Iran.

The results of the present study show that there were significant differences between patients with schizophrenia disorder and healthy controls in seropositivity of* Toxocara*. We replicate the findings of the study conducted by Kaplan et al. [11] and El-Sayed and Ismail [12] that found that* Toxocara* spp. infection is related to schizophrenia disorder. Similarly, Alvarado-Esquivel [13] also reported that* Toxocara* infection is more frequent in patients with schizophrenia.

These results may be related to the fact that patients with schizophrenia have inadequate hygiene and self-care skills, and they have a greater tendency to eat inappropriate things [12]. Considering the correlation between lifestyle and* Toxocara* infection, and that* Toxocara* spp. is common in areas with low hygiene [11], abnormal behaviors and poor personal hygiene observed in patients with schizophrenia expose them to toxocariasis.

We considered gender differences in seroprevalence of* Toxocara* spp. infection. Although anti-toxocariasis IgG antibodies were significantly more prevalent in men with schizophrenia than in healthy men, no difference was found in seroprevalence of* Toxocara* infection between patients and healthy women. This may be related to the role of gender in the experience of illness, treatment, and recovery of schizophrenia disorder. Women with schizophrenia disorder have better global outcomes than men [42]; hence, it is less probable that women with schizophrenia are exposed to toxocariasis.

In the present study, there were no differences in the prevalence of* Toxoplasma* IgG seropositivity between patients with schizophrenia and healthy subjects. The finding is consistent with the result of the study conducted by Saraei-Sahnesaraei et al. [36] and Xiao et al. [37]. However, these results are in contrast to intervention studies [43, 44] and some direct studies [25–31, 34, 35] that support the link between toxoplasmosis and schizophrenia disorder.

The reason for the difference in these findings may relate to the various genotypes of* T. gondii* that vary in prevalence geographically [36] and have distinct neuropathogenic potential [34, 35]. It also may be due to the timing and route of infection, varying degrees of pathogenicity among infecting organisms, or heterogeneous etiology of schizophrenia disorder [45].

There are a number of challenges to these types of epidemiological studies, including the relative insensitivity of some serological assays [46], arbitrary cutoff selection, classification by percentile ranges, grouping results as low, intermediate, or high, and analyzing antibody levels as a continuous rather than dichotomous or categorical variable [27].

In addition, for the purpose of respecting patients' rights, only those who are able and willing to complete the informed consent form are able to participate in the studies. Previous research suggests that clinical symptoms of infected schizophrenic patients are more severe than those of noninfected patients [47], therefore reducing the probability of incorporating infected schizophrenic patients into studies.

In terms of gender difference, we found differences between male and female patients with schizophrenia disorder (*P* = 0.009). Similar results, that is, higher seropositivity in schizophrenic women than in schizophrenic men, were also reported by Dickerson et al. [48].

The present finding is in contrast with the studies conducted by Alvarado-Esquivel et al. [49], Yuksel [50], and Xiao et al. [37], which demonstrated that seroprevalence of anti-*T. gondii* IgG is not different between men and women with schizophrenia disorder. On the other hand, Lindová et al. [51] reported higher seropositivity in male than in female schizophrenic patients.

Higher burdens in women may be explained on the basis of animal studies. Spleens of male mice produce higher level of interferon-gamma (IFN) in the early stages of* Toxoplasma* infection than those of female mice. High levels of IFN and tumor necrosis factor-alpha help male mice to respond to* T. gondii* infection more rapidly and to control the parasite multiplication [52].

Regarding the residential area, some previous studies found that the risks for* Toxocara* and* Toxoplasma* infections are higher in rural areas than in urban areas [50, 53–55]. Our study failed to show any differences between residential areas in the prevalence of infections, consistent with some other reports [12, 37].

Finally we did not find any significant differences in prevalence of* toxoplasmosis* and* toxocariasis* infections across various age subgroups, subtypes of schizophrenia, or between patients with first-episode schizophrenia disorder and those with recurrent episode schizophrenia.

The screening for* Toxocara* spp. antibodies depends on ELISA and Western blotting, which are the most common methods for immunodiagnosis of toxocariasis. Western blotting is more sensitive and specific than other available tests for diagnosing toxocariasis, capable of detecting low levels of* Toxocara* antibodies [56].

## 5. Conclusion

In conclusion, our research suggests that patients with schizophrenia disorder are at an elevated risk for* Toxocara* spp. infection. Moreover, contamination with* T. gondii* should be considered as one of the risk factors for schizophrenia disorder in women.

## Figures and Tables

**Table 1:** Demographic characteristics of the patient and control groups.

Feature	Frequency		
	Patients' group	Control group	Total
*T. gondii *			
Positive	34 (34%)	45 (47.36%)	79 (40.51%)
Negative	66 (66%)	50 (52.63%)	116 (59.48%)
*Toxocara *			
Positive	14 (14%)	4 (4.39%)	18 (9.23%)
Negative	86 (86%)	91 (95.78%)	177 (90.76%)
Sex			
Female	35 (35%)	43 (45.74%)	78 (40.20%)
Male	65 (65%)	51 (54.25%)	116 (59.79%)
Residence			
Urban	76 (76%)	89 (93.98%)	165 (84.61%)
Rural	24 (24%)	6 (6.31%)	30 (15.38%)
Marital status			
Single	64 (64%)	77 (81.05%)	141 (72.30%)
Married	25 (25%)	18 (18.94%)	43 (22.05%)
Divorced/widowed	11 (11%)	0 (0)	11 (5.64%)
Level of education			
Grade school	74 (74%)	0 (0)	74 (37.94%)
12 years/high school	18 (18%)	9 (9.47%)	27 (13.84%)
University degree	8 (8%)	86 (90.52%)	94 (48.20%)
Ethnicity			
Fars	46 (48.42%)	15 (17.85%)	61 (34.07%)
Arab	15 (15.78%)	33 (39.28%)	48 (26.81%)
Lor	19 (20%)	34 (40.47%)	53 (29.60%)
Others	15 (15.78%)	2 (2.38%)	17 (9.49%)
Age (years)			
<20	0 (0)	4 (4.21%)	4 (2.05%)
20–29	32 (32%)	83 (87.36%)	115 (58.97%)
30–39	34 (34%)	7 (7.36%)	41 (21.02%)
40–49	23 (23%)	0 (0)	23 (11.79%)
>50	11 (11%)	1 (1.05%)	12 (6.15%)

**Table 2:** Seropositivity of *Toxoplasma gondii* and *Toxocara* antibodies in patients with schizophrenia disorder and healthy controls.

	Patients' group	Healthy	Sig	OR	CI_95_
	*N* (%)	*N* (%)			
*Toxoplasma gondii *	34/100 (34%)	45/95 (47.36%)	0.08	0.57	0.32–1.02
*Toxocara *	14/100 (14%)	4/95 (4.3%)	0.02	0.27	0.08–0.8

**Table 3:** The distribution of *Toxoplasma gondii* and *Toxocara* antibodies in patients with different subtypes of schizophrenia disorder.

Subtypes of schizophrenia	*T. gondii* *N* (%)	*P* value	*Toxocara* *N* (%)	*P* value
Paranoid type	24/67 (35.82%)	0.9	10/64 (1.56%)	0.5
Catatonic type	3/7 (42.85%)		1/7 (14.28%)	
Residual type	1/3 (33.3%)		0/3 (0)	
Undifferentiated type	6/18 (33.3%)		2/18 (11.11%)	
Disorganized type	0/5 (0%)		1/5 (20%)	

**Table 4:** The distribution of *Toxoplasma gondii* and *Toxocara* antibodies in patients with schizophrenia based on clinical course of treatment.

	Clinical course	*N* (%)	*P* value	OR	CI_95_
*Toxoplasma gondii *	First episode	13/30 (43.33%)	0.1	2.08	0.84–5.12
	Recurrent episodes	18/67 (26.86%)			
*Toxocara *	First episode	8/30 (26.66%)	0.06	0.22	0.06–0.75
	Recurrent episodes	5/30 (16.66%)			

**Table 5:** The seroprevalence of anti-*T. gondii* IgG antibodies in patients with schizophrenia disorder based on gender.

	Patients' group				
	*N* (%)		Sig	OR	CI
	Male	Female			
*Toxoplasma gondii *	16/65 (24.61%)	18/35 (51.42%)	0.009	0.3	0.12–0.73
*Toxocara*	8/65 (12.30%)	6/35 (17.14%)	0.5	1.47	0.46–4.65

**Table 6:** The distribution of *Toxoplasma gondii *and *Toxocara *antibodies in patients with schizophrenia disorder based on their residential area.

	Patients' group				
	*N* (%)		Sig	OR	CI
	Urban	Rural			
*Toxoplasma gondii *	16/76 (21.05%)	4/24 (16.66%)	0.7	1.36	0.39–4.7
*Toxocara *	6/76 (7.89%)	3/24 (12.5%)	0.3	1.7	0.37–7.6

**Table 7:** The distribution of latent toxoplasmosis according to the age in patients with schizophrenia disorder and healthy controls.

	Patients' group	Healthy	Sig	OR	CI_95_
	*N* (%)	*N* (%)			
Age (years)					
<20	0 (0%)	0/4 (0%)			
20–29	10/32 (32.25%)	37/83 (44.57%)	0.1	2.9	0.68–12.51
30–39	14/34 (41.17%)	5/7 (71.42%)	0.1	0.2	0.04–1.5
40–49	6/23 (26.08%)	0/0 (0%)			
>50	4/11 (36.36%)	1/1 (100%)	0.4	0.3	0.1–0.7

**Table 8:** The distribution of toxocariasis according to the age in patients with schizophrenia disorder and healthy controls.

	Patients' group	Healthy	Sig	OR	CI_95_
	*N* (%)	*N* (%)			
Age (years)					
<20	0 (0%)	0 (0%)			
20–29	4/32 (12.5%)	3/83 (4.81%)	0.1	2.9	0.68–12.5
30–39	6/34 (17.14%)	1/7 (14.28%)	0.6	1.2	0.12–12.2
40–49	2/23 (8.69%)	0/0 (0%)			
>50	2/11 (18.18%)	0/1 (0%)	0.8	0.81	0.61–1.08
